# Supplementary material for: Are carbon emissions trading and green financial instruments synergistic? -Comprehensive quantitative research based on content analysis
Source: PLoS One. 2024 Mar 7;19(3):e0298601. doi: 10.1371/journal.pone.0298601 (PMC10919601; doi:10.1371/journal.pone.0298601)
Supplement: S1 Data — (ZIP) [file pone.0298601.s001.zip › Data/地级市Z_F工作报告.zip!/░▓╗╒/╦▐╓▌╩╨/└┤╫╘╣1⁄2╓┌║┼í╛┬φ┐╦╩2╛▌═°í┐_2018.docx]

2018年1月5日在宿州市第五届人民代表大会第一次会议上

市长 杨 军

各位代表：

现在，我代表市人民政府，向大会报告政府工作，请予审议，并请市政协委员和其他列席人员提出意见。

一、2017年和过去五年工作回顾

2017年，在省委、省政府和市委坚强领导下，全市上下认真学习贯彻党的十八大、十九大精神，深入践行新发展理念，坚持稳中求进工作总基调，奋力推进改革发展，较好完成了市四届人大六次会议确定的目标任务。

初步核算，全市生产总值1503.9亿元，增长9.1%；财政收入156.5亿元，增长11.9%；固定资产投资1403亿元，增长10.8%；社会消费品零售总额533.7亿元，增长11.9%；城镇、农村常住居民人均可支配收入27703元和10859元，分别增长8.5%、9.5%。主要经济指标增速好于预期、全省靠前，全市经济稳中有进、稳中向好，社会大局和谐稳定。

产业转型迈出新步伐。新型工业化加速推进。制定实施促进经济平稳健康发展“30条”、推进制造强市建设等系列政策，工业总量和运行质量加速提升。推进“3111”工程突破攻坚，规上工业增加值增长9.8%，县区、园区首位产业产值950亿元、增长15%。深入推进“三重一创”，战略性新兴产业产值、高新技术产业增加值分别增长28.8%、30.4%。实施“1245”重点技改行动，完成投资425亿元，占工业投资58%。项目建设提速提效，开展项目谋划“百日攻坚”，入库重点项目387个，总投资2233亿元；钱营孜低热值煤发电、汉能光伏等项目加快建设，埇桥皖能、灵璧光大垃圾电厂建成运营，绿色家居产业园、海升果酒等项目开工建设。现代服务业持续提升。获批省级服务业集聚区6家、入园企业2000家。实现服务业增加值511.6亿元，增长11.6%，占GDP比重提高4.6个百分点；全市余额存贷比61.2%，新增存贷比90%。埇桥区被确定为“十三五”全省首批服务业综合改革试点单位。四个县全部入选国家电子商务进农村综合示范县，市高新区获评安徽省电子商务示范园区。国购广场建成开业，苏宁广场、绿地高铁小镇开工建设。文化旅游产业热度攀升，运河人家等一批项目建成运营，国内旅游接待人次达2100万，综合收入突破140亿元。全省电商进农村全覆盖工作现场会在砀山县召开。现代农业加快发展。全市规模以上农产品加工产值、农业产业化水平稳居全省第一方阵，农业产业化联合体经营模式被国家六部委联合推广。农村“三变改革”扎实推进。全市20条沿路产业示范带加快建设。奖补“三品一标”企业58家，砀山县获批创建国家有机产品认证示范区，泗县被命名为省质量强县示范县。埇桥区获批创建国家现代农业产业园。萧县循环工业园被认定为国家农业产业化示范基地。泗县、灵璧县获批全省秸秆综合利用试点县。

城乡面貌发生新变化。围绕“一中心、两新区”思路，统筹推进城市规划、建设、管理。规划引领显著增强。编制完成《宿州市主体功能区规划》《大宿城空间战略发展规划》，新一轮《宿州市城市总体规划》修编加快推进。重点工程建设成果丰硕。全市实施重点工程204个，完成投资282.4亿元。“五馆一院一中心”、国道104泗县段、国道310萧县段、符离大道、高铁宿州东站改扩建等项目建设加快，宿州机场、淮宿蚌城际铁路、宿州火车站改造等项目前期工作有序推进。徐明高速灵泗段、泗许高速泗县段全线贯通，郑徐客专、新汴河北堤景观大道引河桥建成通车，国省干线一级公路在建规模达277公里，新汴河复航整治工程取得突破性进展。“两治三改”全面铺开。“棚户区”“城中村”改造新开工41585套、437万平方米，开工率101.5%；基本建成38645套，基本建成率297%，开工率、基本建成率稳居全省前列。控违工作成效显著。县镇建设扎实推进。四个县建设重点工程100个，完成投资153亿元，“555”工程基本完成。萧县凤城新区、泗县北部新城等重点工程进度加快，县城大建设的框架逐步拉开。美丽乡村建设皖北领先。农村环境整治“三大革命”全面启动，完成改厕9.1万户。砀山“酥梨小镇”获批首批省级特色小镇。

脱贫攻坚展现新作为。围绕“1234+产业扶贫”，全市财政统筹整合涉农资金近30亿元支持脱贫攻坚，其中财政专项扶贫资金8.7亿元。全市11.68万贫困人口、104个贫困村有望脱贫出列，贫困发生率降至2.89%。脱贫攻坚合力增强。健全扶贫层级管理体系，市级领导分片联系县区，扶贫队长、扶贫专干、扶贫小组长实现村级全覆盖。基层基础更加扎实。开展精准扶贫“质量再提升”“重精准、补短板、促攻坚”专项整改、建档立卡贫困人口动态调整等活动，做到应进则进、应出则出。保障政策提标扩面。教育扶贫实现资助全覆盖。在全省率先出台综合医疗保障政策、率先全面实施“一站式”即时结算、率先兑现慢性病“180”补充医疗保障政策，在全国健康扶贫现场会上交流经验，全省县域结对帮扶推进脱贫攻坚现场会、健康脱贫现场会在我市召开，泗县获评全国健康扶贫工程示范县。农村低保标准提高到每人每年3800元，实现低保线与扶贫线“两线合一”。十大工程稳步实施。产业扶贫实现到村项目全覆盖；光伏扶贫超额完成省下达任务；扶贫小额信贷总量全省靠前；危房改造三年任务一年完成；农村道路畅通工程完工2206公里，全省第一；电商扶贫成效显著，全国电商扶贫会议推介砀山经验；大型扶贫现代戏《春风化雨》赴省演出。

生态文明呈现新气象。大气质量持续改善。狠抓扬尘、货运车辆、燃煤锅炉、油烟废气等问题治理，完成255家采石矿山扬尘控制、43个非煤矿山地质环境治理，主城区燃煤小锅炉实现“清零”，完成节能减排目标任务。蕲县码头、矿区周边、非煤矿山等一批大气污染源得到治理。水环境治理扎实推进。划定饮用水水源地范围及各级保护区。提前完成省政府下达的2017年黑臭水体治理任务，立牌监测全市102个入河排污口，建立推行三级河长制。建成工业集中区污水处理厂8个。生态建设得到加强。开展林业增绿增效行动，推行林长制，成片造林11.8万亩，新增提升绿地812万平方米，市场化养护管理公共绿地450万平方米。大力推进土地开发复垦整理，完成补充耕地2.2万亩，位居全省第一。统筹推进农村生态环境保护，44个村庄环境综合整治全面完成。

改革开放释放新活力。供给侧结构性改革进展顺利，“去降补”任务落地见效。“放管服”改革持续深化。建立完善市级权力清单、责任清单、公共服务清单、中介服务清单和涉企收费清单，“互联网+政务服务”平台实现与省平台互联互通，市政务服务中心被评为全国行政服务大厅典型案例“百优十佳”单位。公共资源交易基本实现全流程电子化。商事制度改革深入推进。实行“多证合一、一照一码”，新增企业11212户，新登记个体工商户29225户，商标注册申请量首次突破万件。金融创新有声有色。续贷过桥、“税融通”、新型政银担等投放资金189亿元；新宇药业实现主板报审，新增新三板挂牌企业3家，新设10支产业发展基金。科技创新实现突破。研发投入增幅全省第一，高新技术企业增至85家，建成国家级工程技术研发中心1家、省级创新创业平台24家；全省首家石墨烯领域工程实验室和安徽省裸眼3D显示工程实验室获批，省级高层次科技人才团队实现“零突破”。全省开发区首家博士交流创业中心在市高新区揭牌成立。重点领域改革不断破题。新实施一批基础设施和公共服务PPP项目，国有土地二级市场改革试点、供销社综合改革试点稳步推进，财税金融、国资国企、医药卫生等重点改革扎实有序。全面深化改革第三方评估工作在全国推广。对外开放走向纵深。埇桥区、泗县获评全国中小城市投资潜力百强；砀山果蔬出口质量安全示范区、泗县服装外贸转型基地加快建设；皖北保税仓基本建成；市经开区、宿马园区荣获“中国产业园区成长力百强”，市高新区荣获“中国产业园区创新力百强”，鞋城获评中国轻工业特色区域和产业集群管理服务先进单位；第一届国际云安全大会成功举办；全省招商引资工作现场会在萧县召开。

公共服务得到新提升。民生保障力度持续加大，财政民生支出295亿元，占财政总支出86%。就业社保体系不断完善。新增就业4.7万人，城镇登记失业率3%；实施全民参保计划，推进机关事业单位养老保险制度改革，企业退休人员基本养老金人均月增132元；新增养老床位2000余张。教育体育事业蓬勃发展。基础教育协调发展，新建、改扩建公办幼儿园43所，维修改造校舍57.9万平方米，义务教育发展基本均衡县实现全覆盖；职业教育改革深化，职教园区初具规模，获得全国职业技能大赛金奖2项。成功举办宿州国际半程马拉松赛和砀山国际马术耐力赛。卫生计生工作持续加强。启动实施“411”计划，市立医院儿科病区投入使用，新建乡镇卫生院8所、村卫生室146个；积极推行“四型”医院创建，人才队伍“三大计划”全面实施，重点人群家庭医生签约率达70.9%，学科建设实现省级优先发展重点专科“零突破”，医疗服务体系建设取得重大进展，一批县级综合医院及中医院投入使用；埇桥区被认定为第四批国家慢性病综合防控示范区；全面两孩政策有效落实，计生队伍转型成效显著，公共卫生事业继续加强。社会治理不断创新。“落实企业安全生产主体责任推进年”活动深入开展，十九大维稳安保任务圆满完成，应急管理体系不断完善，食品药品监管规范有序，埇桥区成功创建全省食品安全示范区，“平安宿州”建设卓有成效。获批创建国家公交都市，新增纯电动公交车120辆。社会信用建设全面推动。“七五”普法活动深入开展。圆满完成第三次全国农业普查。人民防空工作成绩显著。民族宗教、防震减灾、外事侨务、物价、对台、气象、档案、地方志、妇女儿童、残疾人等事业不断进步。

各位代表！过去一年目标任务的顺利完成，标志着本届政府五年任期的圆满收官。五年来，我们积极应对宏观经济形势变化，砥砺奋进，攻坚克难，取得了令人鼓舞的发展成就，较好完成了“十二五”规划任务，稳步迈入“十三五”承上启下的发展阶段。

这五年，我们坚持产业引领加快发展，综合实力显著增强。全市生产总值连跨5个百亿元台阶，年均增长9.4%。财政收入连跨7个十亿元台阶，年均增长13.5%，是五年前的近2倍。首位产业不断壮大，规上工业增加值年均增长10.7%。战略性新兴产业产值突破百亿元，年均增长31%；宿州成为全国第五个量子通信节点城市。固定资产投资年均增长18%，是五年前的2.3倍。一产比重降至20%以下，实现历史性突破。服务业增加值增幅连续五年全省靠前。社会消费品零售总额累计2082亿元，连续五年保持两位数增长。现代农业“两区”建设走在全国前列，粮食生产实现“十四连丰”，农民专业合作社发展到11465家，市级以上龙头企业635家，农业产业化联合体模式连续三年写入省委1号文件并上升到国家层面推广。园区转型升级步伐加快，全市开发区核准面积从69.4平方公里增加到150.5平方公里，开发区承载能力显著增强。县域经济发展提速，占全市经济比重57%以上，四县一区固定资产投资、财政收入占全市比重分别达84.5%、66.8%。埇桥区、泗县入选全国农民工返乡创业试点地区。

这五年，我们坚持加快基础设施建设，城乡面貌焕然一新。累计实施重点工程1025个，累计投入1150亿元，相继建成新汴河景观带、宿灵泗快速通道等一批标志性工程。不断扩展城市外延，大外环全线贯通，规划区面积超过200平方公里，主城区实现双“85”目标。持续提升城市内涵，成功获批国家首批电信普遍服务试点城市、“宽带中国”示范城市，“城管服务超市”荣获全国创新社会治理最佳案例奖。砀山县、萧县通高铁，全市县县通高速，一级公路里程突破350公里，交通建设连续三年全省前列。县城建设提质扩容，特色城镇亮点纷呈，美丽乡村建设连续四年全省前列，建成省级示范中心村112个。扎实开展“16城”同创，荣获“国家园林城市”称号，全市森林覆盖率达30.9%。蝉联“安徽省文明城市”，四县一区全部入围省级文明县城、文明城区，城市知名度和美誉度不断提升。

这五年，我们坚持深化改革全面开放，创新活力空前迸发。打好深化改革“组合拳”，重要领域和关键环节改革取得重大突破。持续推进“放管服”改革，“三集中、三到位”全面落实，市县乡村四位一体的“互联网+政务服务”平台投入运营，行政审批类、公共服务类项目“网上可办”，持续打造“四最”营商环境。累计新增登记注册企业43178户，民营经济增加值占GDP比重65.9%。深入推进户籍制度改革，农村转移人口28.1万人。文化、医药卫生等体制改革有序推进，人地挂钩改革试点、“劝耕贷”等走在全国前列，不动产登记工作扎实开展。流转土地413.2万亩，流转率46.6%。持续落实一系列招商制度，累计到位内资5470亿元，实际利用外资32亿美元，进出口总额累计29.6亿美元。宿州海关正式开关，出入境检验检疫局挂牌开检。获评全国社区侨务工作、为侨服务工作示范单位，新增国际友城和友好交流城市6对，对外开放的“朋友圈”越来越广。

这五年，我们坚持社会事业共建共享，民生福祉大幅提升。深入贯彻以人民为中心的发展思想，着力补齐教育、卫生、文化等事业短板，提升基本公共服务均等化水平，人民群众获得感进一步增强。连续五年实施33项民生工程和10件惠民实事，惠及城乡500多万居民。坚持以脱贫攻坚统揽发展全局，预计全市贫困人口从61.3万人减至16.1万人，贫困村从359个减至115个，贫困发生率从11.8%降至3.2%。萧县被评为全国社会扶贫先进县。文化事业、文化产业连结硕果，《石乡党魂——柯增华》等荣获省“五个一工程”奖，建成博物馆、公共图书馆等17座，建成乡镇综合文化站102个。宿州国际网球公开赛被评为全省十佳体育品牌赛事，我市成为全省笼式足球普及提高唯一试点市，竞技体育获国际性、全国性比赛冠军14项、亚军2项。获评全国道德模范及提名奖6人、中国好人27人，涌现出全国道德模范许启金、彭伟平、全国扶贫先进工作者李红玲、全国脱贫攻坚奋进奖获得者李娟等一批先进代表。社会保障体系更加健全，城乡基本养老、最低生活保障、医疗救助、特困人员供养水平大幅提高，城乡居民社会养老保险实现全覆盖。人口计生工作连续四年皖北第一、全省先进。“铸安”行动深入开展，安全事故起数逐年下降。综治工作连续四年受到省委、省政府表彰，信访工作目标考核连续两年全省第一，灵璧县连续三届被评为全国平安县。深化巩固军政军民团结，全国双拥模范城、全国无偿献血先进城市分别实现七连冠、八连冠，国防动员和后备力量建设绩效考评全省第三。新乡贤治理模式获得中央改革办充分肯定。宿州市连续三年获评全国防震减灾工作综合考核先进单位。市统计局荣获全国统计系统先进集体。食品药品安全工作跨入全省第一方阵。

这五年，我们坚持全面推进自身建设，政府工作持续加强。先后开展党的群众路线教育、“三严三实”专题教育、“两学一做”学习教育、“讲重作”专题教育，始终同以习近平同志为核心的党中央保持高度一致，“四个意识”不断增强。深入贯彻习近平新时代中国特色社会主义思想，积极更新理念、转变作风。持续建设法治政府，合法性审查机制市县乡三级全覆盖，行政执法规范化水平显著提升，行政争议得到依法有效化解。自觉接受人大及其常委会监督、政协民主监督，累计办理人大代表议案和建议532件、政协委员提案1285件，满意度分别达到99.4%、99.8%。畅通群众联系渠道，12345政府热线服务平台上线试运行，受理群众诉求10256件，办结率95.6%以上。强化行政监察，推进审计监督、制度规范全覆盖，自觉遵守中央八项规定精神和省、市“30条”要求，不敢腐的目标初步实现，不能腐的笼子越扎越紧，不想腐的堤坝正在构筑。

我们也清醒地看到，宿州依然存在一些发展中的问题：技术创新能力不足，产业转型层次不高，新型工业化动能不强，战略性新兴产业总量偏小；城镇化水平较低，中心城区辐射力弱，城乡发展不均衡；产业结构不优，一产比重依然较大，农业现代化水平不高，三次产业融合不够；发展不平衡、不充分，社会保障、公共服务与人民对美好生活需要还有差距；长期以来自然资源资产管理粗放，环境突出问题较多；脱贫攻坚任务依然艰巨；部分干部执政水平、担当能力有待提高，等等。对此，我们必须强化责任担当，着力加以解决。

各位代表，综观过去五年，我们始终坚定发展信心，不断厚植发展优势，走出了一条增长与转型良性互动、发展与生态相得益彰、经济与社会协调并进的宿州之路，宿州的经济总量、城市能级、区域地位和城市影响力大幅提升，在全省加快发展的大格局中扮演着愈来愈重要的角色。今天的宿州，已经站在蓄势待发、后来居上的新起点，正在开启加速前进、跨越发展的新局面，即将迎来奋力崛起、全面跃升的新时代！

这些发展成就，是市委坚强领导、统揽全局的结果，是历届班子矢志不移、持续接力的结果，是全市人民凝心聚力、顽强拼搏的结果。在此，我代表市人民政府，向奋战在各行各业的全体劳动者，向离退休老同志，向各民主党派、工商联和人民团体，向中央和省驻宿单位、人民解放军驻宿部队、武警官兵、公安干警，向参与宿州建设的海内外企业家和客商、广大援宿干部，以及支持宿州发展的各界人士，表示衷心的感谢！

二、未来五年发展谋划

未来五年，处于“两个一百年”奋斗目标的历史交汇期，也是全面建成小康社会的关键决胜期。党的十九大为我们指明了奋进方向，注入了新的动力。经过多年努力，我市经济转型升级成效初显，综合实力大幅提升，发展基础更加稳固，深化改革红利持续释放，争先进位、跨越发展的势头更趋强劲。尽管前进道路上还有许多风险挑战，但宏观经济长期向好的基本面没有改变，依然蕴藏着前所未有的发展机遇。

随着“一带一路”、中原城市群、乡村振兴等战略规划的实施，振兴皖北政策的有力推进，我们将充分发挥政策叠加的支撑优势，持续不断发力。宿州四级物流网络基本形成、社会消费品零售总额连创新高、一批“宿州云”知名品牌相继入驻，我们将充分发挥多元要素集聚的现实优势，催生一个活力迸发的宿州商圈。传统产业脱胎换骨，战略性新兴产业雨后春笋，高新产业异军突起，“中国云都”崭露头角，我们将充分发挥新型工业化的后发优势，加速实现弯道超车。宿州正处于深度城镇化的窗口期，城镇化率还有很大提升空间，我们将瞄准国家投资方向，充分发挥深度城镇化的潜在优势，加快在基础设施和公共服务短板领域形成一批有效投资，培育新的经济增长点。宿州作为全国重要农产品供应基地和集散中心，农业产业化多年领跑全省，农业“两区”建设方兴未艾，我们将充分发挥农业现代化的比较优势，加速提升现代农业核心竞争力。

基于上述优势，只要全市上下坚定信心，保持定力，主动作为，就一定能够群策群力，众志成城，持续推动经济社会发展不断迈上新台阶。

未来五年，我们将坚定不移地以习近平新时代中国特色社会主义思想为指导，全面贯彻落实党的十九大精神，加强党对经济工作的领导，坚持稳中求进工作总基调，统筹推进“五位一体”总体布局和协调推进“四个全面”战略布局，坚持以新发展理念为统领，以人民为中心，以高质量发展为根本要求，以供给侧结构性改革为主线，以五大发展行动计划为总抓手，深入实施新型工业化、深度城镇化、农业现代化“三大战略”，全力打好防范化解重大风险、脱贫攻坚、环境保护“三大战役”，全面推动经济发展质量、效率、动力“三大变革”，实现更高质量、更有效率、更加公平、更可持续的发展，不断满足人民群众对美好生活的向往，奋力开创现代化五大发展美好宿州建设的新局面！

未来五年，我们将坚持以建设现代化经济体系为战略目标，加快建设实体经济、科技创新、现代金融、人力资源协同发展的产业体系，转方式、调结构、强动能取得突破性进展，实现主要指标增幅、发展质量效益全省前列、皖北领先，全市生产总值突破2200亿元，人均生产总值与全省差距明显缩小，生产总值、财政收入增速超过全省平均水平，经济总量跻身全省第一方阵，综合财力进入全省前八，县区全部实现稳定脱贫，确保与全国全省同步全面建成小康社会。经过努力，基本建成全省新型城镇化和工业化集聚发展创新区、淮海经济区生态文明建设样板区、全国重要云计算大数据产业基地、皖北开放新高地、省际交汇区域中心城市。

深入实施创新发展行动，加快推进产业转型升级。全面实施创新驱动发展战略，拓展发展新空间，打造现代产业体系，提升综合经济实力。持续推进工业强市兴县，做大做强首位产业，促进三次产业融合发展。全面推进制造强市战略，推动互联网、大数据、人工智能与实体经济、传统产业深度融合，加快军民融合产业发展，促进制造业从制造、创造向智造转变。构建发展新体制，搭建创业创新平台，推动大众创业、万众创新。拓展数字经济成长空间，加快国家智慧城市、智慧农业试点建设，培育一批“宿州云”知名品牌。推进“互联网+”行动，构筑发展新动能。创新金融服务和监管体系，守住不发生系统性金融风险底线，增强服务实体经济能力。实行更加积极、有效、开放的人才政策，让各类人才迸发创造活力，为宿州发展贡献聪明才智。

深入实施协调发展行动，构建城乡统筹共进格局。以建设省际交汇区域中心城市为目标，以“大宿城”为中心、4个县城为副中心、萧砀小城镇集群和灵泗小城镇集群为基础，加快构建功能定位清晰、片区布局合理、协同互动发展的现代城镇体系。强化城市片区分工协作，优化产业和功能布局，推进产城融合发展，支持市高新区发展云计算、文化创意、智能制造，市经开区和鞋城发展生物医药、绿色化工、装备制造、鞋服制造，宿马园区发展绿色食品、装备制造、新能源新材料等重点产业。振兴县域经济，大力发展砀山县绿色果蔬食品、农副产品深加工，萧县新型建材、精细化工，埇桥区家居板材、纺织服装，灵璧县机械电子、新材料，泗县机械电子、品牌服装等主导产业。实施乡村振兴战略，制定乡村振兴规划，健全城乡融合发展体制和政策体系，加快构建现代农业产业体系；加快美丽乡村建设，开展好农村环境整治“三大革命”。

深入实施绿色发展行动，不断加快生态文明进程。牢固树立绿水青山就是金山银山的理念，坚持节约资源和保护环境的基本国策。加快补齐生态建设短板，建设“天蓝、地绿、水净”的美丽宿州。全面落实河长制、林长制，深入实施大气、水、土壤、畜禽养殖污染防治行动计划，继续实施林业增绿增效行动，着力改善生态环境。加强生态文明制度建设，严守生态红线，到2020年全面落实主体功能区战略和制度。实行最严格的水资源管理制度、节约用地制度、耕地保护制度，推进节能降耗，大力发展循环经济，到2022年所有乡镇建成污水处理设施，工业固体废弃物综合利用率达95%左右，城市生活垃圾基本实现无害化处理。实行最严格的生态环境保护制度，加大环境治理力度，挥发性有机物排放总量下降10%以上。

深入实施开放发展行动，打造多元要素融通高地。深度融入“一带一路”“京津冀协同发展”“长江经济带”发展战略，主动衔接中原城市群建设，全面参与淮河生态经济带和淮海经济区核心区合作发展，加强与徐州对接连通、融合发展。深化南北合作，推进结对共建园区建设。优化招商引资环境，大力发展外向型经济，打造皖北开放新高地。强化大通道、大平台、大通关建设，加快基础设施互联互通，实现与周边市县全面快捷的道路连通，加强口岸和特殊监管场所建设，加快建设内陆无水港，积极推进宿州民航机场、城市轨道交通、连霍高速宿州段扩容改造工程和宿固蚌、徐淮阜高速公路宿州段建设，协调推进合青高铁宿州段、萧徐轨道交通建设。优化创业环境，激发和保护企业家精神，鼓励创业富民，营造海纳百川、多元融合的创业氛围，凝聚各方力量助推宿州发展。

深入实施共享发展行动，持续增进人民群众福祉。坚持以人民为中心，始终把人民对美好生活的向往作为奋斗目标。坚决打赢脱贫攻坚战，全面实现“户脱贫、村出列、县摘帽”。确保城乡居民收入增速快于经济增长，达到全省平均水平，居民生活质量普遍提高。千方百计稳岗增岗，促进就业创业。加快培养高素质和技能型人才，推动“民工大市”向“技工大市”转变。加强精神文明建设，提高公民素质，争创全国文明城市。深化文化体制改革，培育文化创意、游戏动漫等新型文化业态。完善公共文化服务，加快建设公共文化设施。深化教育改革，推动城乡教育一体化，促进义务教育优质均衡发展，创造普及高中教育条件；支持宿州学院、宿州职业技术学院和皖北卫生职业学院等院校提升综合办学实力和创新能力，加快市县区中职学校建设，提升职业教育整体水平；支持和规范民办教育发展，办好特殊教育；提升教育信息化水平。推进健康宿州建设，加大对市级和县级新建城区、各级开发区医疗资源配置，全面提升二级医院医疗能力，争创3个三级医院，全市乡镇卫生院、社区卫生服务机构、村卫生室等标准化建设实现全覆盖，健全重大疾病防控、预防接种、传染病防治、慢性病管理、妇幼保健等公共卫生服务体系。优先发展公共交通，着力实施城乡客运一体化。健全体育、养老、残疾人等公共服务体系，织牢公共安全保障网，完善社会保障，兜住民生底线，实现全体居民共同迈入全面小康社会。

三、开局之年工作安排

今年是全面贯彻落实党的十九大精神的起步之年，是改革开放40周年，也是新一届政府的开局之年。我们必须坚持以习近平新时代中国特色社会主义经济思想为指导，深入贯彻落实中央和省委、市委经济工作会议精神，按照市委五届四次全会决策部署，统筹推进稳增长、促改革、调结构、惠民生、防风险各项工作，促进经济社会持续健康发展，为全面建成小康社会夯实基础。

综合考虑，今年经济社会发展主要预期目标是：全市生产总值增长8.2%左右，财政收入增速高于经济增长，规上工业增加值增长8.5%左右，固定资产投资增长9.5%左右，社会消费品零售总额增长10.5%左右，城镇常住居民人均可支配收入增长8%左右，农村常住居民人均可支配收入增长8.5%以上，城镇调查失业率控制在5%以内，节能减排完成年度任务，主要动态指标位居全省前列，努力实现本届政府良好开局。

（一）着力转换增长动能，加快构建现代化产业体系。深入落实供给侧结构性改革举措，优化经济结构、转换增长动力，加快构建创新型现代产业体系，增强经济创新力和竞争力。

加快推进新型工业化。强化项目带动，重点项目储备规模超过2500亿元，建设亿元以上重点项目超过600个。细化工业强市措施，深入推进“3111”工程突破攻坚，完成“3个10”目标任务。系统推进“全创改”，加快建设技术和产业、平台和企业、金融和资本、制度和政策四个创新体系，打造贯通产业链、创新链、资金链的创新生态系统。全面实施“三重一创”，做大做强省级云计算战略性新兴产业基地，高标准建设市级战略性新兴产业基地，积极培育精准医疗、生物制药等一批重大新兴产业，培育石墨烯、大健康等一批重大新兴产业专项，推进石墨烯复合功能薄膜新材料、裸眼3D显示、医疗大数据等省级重大创新平台建设，战略性新兴产业产值增长20%。大力发展高新产业，高新企业达到90家，高新产业增加值增长15%。以智慧经济和创业型经济为引领，开展新一轮技术改造，实施制造强市“1525”行动，全面提升轻纺鞋服、化工建材等传统产业，推进重点技改项目200个，技改投资增长8%以上。开展质量品牌提升行动，创建国家质量强市示范城市，加大名牌产品培育力度，高标准评选推荐政府质量奖，培育省名牌产品5个以上。

提升现代服务业。大力发展现代物流、总部经济、健康养老等现代服务业，扩大移动互联网、物联网等信息消费，加快汴河文化旅游开发等重点项目建设，创建省级服务业集聚区1家、示范园区1家。建设“电商宿州”，加强与国内外知名电商战略合作，加快电商产业园、跨境电商综合服务平台建设，发展“互联网+快递”新兴消费，推进电商进农村全覆盖。积极构建全域旅游格局，将旅游业培育成为重要支柱产业。实施旅游强市“五个一批”工程，推进皇藏峪5A级景区和新汴河景区、钟馗文化园、运河人家4A级景区创建，加快符离生态旅游大道沿线景点规划建设。

振兴县域经济。推动县域经济转型发展，支持融入周边中心城市产业链布局和创新体系。实施“3111”工程，做强各县区首位产业，大力发展特色经济、开放型经济，不断优化县域经济结构。加大“一县一策”专项扶持力度，强化人才、金融、用地等对县域经济的支撑作用，培育壮大企业家队伍。积极争取新设市辖行政区，支持萧县等有条件的县撤县设市。

促进园区创新发展。深化园区体制机制改革，推动开发区优化整合，引导开发区特色化、差异化发展。支持市经开区争创国家级经开区，支持宿马园区深化南北合作共建，支持市高新区打造辐射全国的云计算产业集群，支持鞋城打造区域最具特色的现代制鞋产业聚集区。推动宿马高铁小镇、市经开区生物医药科技园等重点项目建设。提升县域开发园区发展水平，推动张江萧县高科技园上升为沪皖合作战略平台，加快埇桥绿色家居产业园、泗涂现代产业园等园区建设。支持埇桥经开区与徐州对接合作。加快产城融合发展，完善园区基础设施，加强双创空间、科技孵化器、标准化厂房等平台建设，提升园区承载功能。

（二）深入实施深度城镇化，加速提升城乡承载能力。全面推进以人为核心的新型城镇化，加强城乡一体化建设，全市常住人口城镇化率增长1.5%以上。

提升主城区城市能级。完善城市规划体系，改革规划管控体系，全面推进“多规合一”，加快土地利用总体规划、新一轮城市总体规划修编。推动汴北新区、城东新区建设，实施四大类120项重点工程，总投资179亿元，主城区实现“双90”目标。实施“两治三改”专项行动，构建征迁、安置、开发三位一体的闭合系统，主城区及各县城老旧小区改造工作三年完成。加大棚户区改造力度，加快保障房、公租房建设，全面完成棚改三年计划任务。完成符离大道道路主体工程，启动新汴河直通洪泽湖水上航道工程项目，开工建设宿城银河一路、胜利路、纺织路东西大动脉路网工程，完成宿城西大外环路网工程，全面启动老火车站综合改造工程，确保老城区地下管线智能化改造、汴北新城区地下管廊、城市小区供热试点工作取得实效。新建和改造提升一批标准化菜市场、街头游园、绿带水系、公共厕所。探索名校办分校方式，推动市区优质教育资源扩容，新建、改扩建中小学10所，三年内主城区建成公立幼儿园30个。深化城管体制改革，提高城管精细化水平。动员全市上下以“16城”同创为抓手，在争创“全国文明城市”工作中取得突破性进展。

促进县乡提质扩容。编制县域乡村建设规划，优化镇村布局，引导调控县城、镇村合理发展。创新城乡建设用地增减挂钩机制，探索就近就地的城镇化新模式，提高城镇吸引力和容纳力。加强公共基础设施建设，大力实施100项重点工程，总投资179亿元。持续实施县域建设“555”工程，强化乡镇环境综合整治，着力建设宜居县城、美丽乡村，创建省级特色小镇1个、市级特色小镇2个。深化户籍制度改革，积极落实常住人口和农业转移人口市民化激励机制。

完善基础设施。加快城市轨道交通、宿州机场、四个县通用机场前期工作，完善国省干线公路快速通道网络，推动淮宿蚌城际铁路、合青高铁宿州段等加快建设，建成使用“五馆一院一中心”，不断完善城市配套功能。加强水利基础设施建设，新开工建设怀洪新河水系洼地治理工程。合理规划布局电动汽车充电基础设施，加快新能源汽车推广应用。

（三）大力推进乡村振兴战略，融合促进农业现代化。着力转变农业发展方式，以打造“两区一园”为抓手，加快推进农业农村农民现代化，推动农业大市向农业强市跨越。

加快发展现代农业。创新实施“1234”战略，推进农业绿色发展，加快推进“百千万”现代农业示范工程，高水平建设创新型农业现代化产业园。按照提单产、稳总产、调结构、强品质的要求，保障粮食生产安全。完善龙头企业、合作社与农户的利益联结机制。推进多种形式适度规模经营，加快新型农业经营主体培育，推动埇桥区国家现代农业示范区建设取得新突破，鼓励每个县区选择5个村整体流转土地，农业产业化联合体发展到280家，新增市级以上示范家庭农场、农民专业合作社示范社各50家。招大、引强、奖优农业龙头企业，鼓励宿州帝元等企业创新发展循环农业。大力发展“三品一标”农产品，集中打造区域特色明显、布局集中连片果蔬基地50个，市级以上龙头企业超660家，农产品加工产值保持全省前三。深化农村土地制度改革，开展“三变”改革试点，完善农村土地“三权”分置办法，积极探索确权登记颁证成果运用。

推进美丽乡村建设。加强中心村和新型农村社区建设，支持泗县试点全省美丽乡村建设整县推进工作，启动建设2018年45个省级示范中心村。大力发展乡村旅游和休闲农业，加快推进乡村旅游富民工程、乡村旅游示范村、高星级农家乐建设。结合空心村整治，开展农村人居环境整治行动，深入推进农村垃圾、污水、厕所专项整治“三大革命”，持续改善农村人居环境。深入推进“四好农村路”建设，为加快推进农业农村现代化提供更好保障。大力实施高标准农田建设项目，加快推进农村土地综合整治。建成淮水北调及市级配水工程，夯实农业生产基础。

拓宽农民增收渠道。深化农村集体产权制度改革，建成并规范运转县区级农村产权流转交易中心。推进“劝耕贷”工作。实施新型农业经营主体带头人培育行动，积极培育新型职业农民和农业职业经理人，支持和鼓励农民就业创业。完善龙头企业带动农户的利益联结关系，保障农民财产权益。

（四）聚焦精准精细扶贫，坚决打赢脱贫攻坚战。坚持精准扶贫、精准脱贫，按照“1234+产业扶贫”要求，提升脱贫质量，健全长效机制，全面实现三县一区脱贫摘帽、115个贫困村出列、11.93万贫困人口脱贫。

持续打牢基层基础。完善贫困户信息数据核准核实机制，提升贫困人口识别精准度。落实精准帮扶措施，做到帮扶成效到村到户到人。加大非贫困村和自然村扶贫力量，切实解决非贫困户特别是“边缘户”的帮扶问题。深入落实“单位包村、干部包户”工作制度。加强扶贫对象动态管理，建立健全返贫预警机制，及时掌握返贫情况，落实返贫户和新增贫困户帮扶措施，确保政策落实、支持力度、包保责任不变。

深入推进政策落实。精准谋划实施产业扶贫工程，巩固提升光伏发电、乡村旅游、农村电商等扶贫成果，大力培育特色产业。推广“四带一自”产业扶贫模式，完善产业扶贫利益联结机制，大力发展资产收益扶贫，加快实现产业扶贫全覆盖。深入落实教育扶贫、健康脱贫、兜底脱贫等政策，适度增加重点人群特惠政策，打好政策补丁，打通政策落地“最后一公里”。全面梳理扶贫项目，动态管理县级扶贫项目库、乡村扶贫项目清单。建立小额信贷使用监管机制，规范发展扶贫小额信贷。加快扶贫项目建设，强化资金监管，提升使用效益。

注重激发内生动力。拓宽就业扶贫渠道，大力推广扶贫工厂、就业扶贫驿站，探索创新多种模式，让更多贫困户就近就业增收。激励和约束贫困老人子女履行赡养义务。持续聚力扶志和扶智，强化贫困群众素质教育和技能培训，逐步增强“造血”功能，引导贫困户树立信心、勤劳脱贫。

强力聚焦深度贫困。加大要素投入力度，推动新增脱贫攻坚资金、项目、举措向深度贫困县、村、户倾斜。重点实施贫困村提升工程，支持深度贫困村发展特色产业、壮大集体经济、强化公共基础设施建设。

（五）加强生态文明建设，积极拓展绿色发展空间。坚持节约优先、保护优先、自然恢复的方针，落实主体功能区战略和制度，突出抓好环境治理，切实改善生态环境。

解决突出环境问题。落实大气、水、土壤三个“10条”，开展城乡黑臭水体治理、集中养殖粪污资源化利用、秸秆综合利用提升三个专项行动，强化畜禽养殖污染防治，建立完善大气、水环境监测网络体系，持续抓好环保突出问题整改。加强扬尘污染防治、机动车尾气治理、餐饮油烟整治、企业高架污染源管控，加快推进砖瓦窑厂治理、非煤矿山整治，打赢蓝天保卫战。完成工业集聚区污水集中处理设施、城市黑臭水体治理、饮用水水源地保护等项目建设，打好碧水攻坚战。加强农业面源污染防治，按照规范化、规模化、生态化发展养殖业，创建畜禽养殖标准化示范场省部级3个、市级5个，畜禽养殖废弃物综合利用示范场16个。加大秸秆禁烧监管力度，持续提升秸秆综合利用水平。

推进绿色发展。开展工业、重点产业、建筑等领域能效提升行动，壮大节能环保、清洁生产、清洁能源等产业。建立市场导向的绿色技术创新体系，加快建设绿色建筑，优先发展公共交通，大力推进秸秆综合利用。开展创建节约型机关、绿色家庭、绿色学校、绿色社区等行动，推进资源全面节约、循环利用。

加强生态系统保护。以“两河一廊”三大生态板块建设为引领，统筹推进山水林田湖草生态保护与修复工程，加快采煤沉陷区综合治理，新增和提升绿化面积550万平方米，新建绿道46公里，划定生态保护红线、永久基本农田、城镇开发边界三条控制线。健全市县乡三级河长制、市县乡村四级林长制责任体系。认真落实生态环境损害赔偿制度改革方案，完善生态环境管理和损害评估制度，建立生态环境损害责任终身追究制度，实行领导干部自然资源资产离任审计，健全生态环保督察制度，严厉制止和惩处破坏生态环境行为，保护生态环境和人民环境权益。

（六）强化改革创新引领，厚植开放经济竞争优势。聚焦构建发展新体制，深化经济体制和行政体制改革，全面形成上下互动、内外联动的开放格局。

扎实推进“破、立、降”。推进重点行业、重点企业脱困发展和转型升级，全面完成省下达的淘汰落后产能任务，配合做好“三供一业”分离移交。加大非住宅商品房去库存力度，扩大公租房货币化保障，加快建立租购并举的住房制度。继续落实全面营改增和各项减税降费政策，降低交易成本和要素成本。加强金融风险和政府性债务风险防控。

推进重点领域改革。开展外商投资企业属地登记管理制度改革。深入推进“放管服”改革，推动政府权力清单、责任清单、涉企收费清单、公共服务清单、中介服务清单落地见效，完善“多证合一、一照一码”登记制度。深化科技体制改革，加快建立技术创新体系，促进科技成果转化。健全投融资体制，规范实施PPP模式，发挥投资引领发展的杠杆作用。深入实施金融服务创新推进工程，健全地方金融监管体系，新增贷款200亿元，主板上市在审1家，新增新三板挂牌企业3家，直接融资50亿元。深化国资国企改革，发展混合所有制经济，推动国有资本做优做强，有效防止国有资本流失。深入推进价格机制、统计管理制度改革。

着力扩大对外开放。主动参与中原城市群、淮河生态经济带、淮海经济区核心区一体化建设，积极对接徐州都市圈。申报建设保税物流中心，进出口增幅高于全省平均水平。推进战略招商、产业招商、专业招商、以商招商、委托招商，落实招商引资“1+5”政策文件，实际到位内资增长9%，实际利用外资增长7%以上，民营经济增加值占GDP比重67%左右。强化引智引技，推进人才创业扶持计划和产业领军人才等重大人才项目，引进一批海内外高层次人才就业创业。

（七）完善社会治理格局，着力营造和谐发展环境。坚守底线、完善制度、引导预期，打造共建共治共享的社会治理格局。

筑牢安全生产防线。扎实推进安全生产领域改革发展，持续深化依法治安实践，推进“铸安”行动全覆盖，坚决遏制重特大安全事故。健全安全生产责任制，持续推进企业落实安全生产主体责任，加强安全生产执法监管队伍建设，保持安全生产形势持续稳定。

健全公共安全体系。实施食品安全战略，加强食品药品安全监管，完成104个基层监管所标准化建设，争创省级食品药品安全城市。深化民兵力量改革，提升国防动员和后备力量建设水平，加强人民防空建设，推进军民深度融合发展，做好优抚、安置工作，巩固双拥模范城创建成果，促进社会总体安全。加强社区建设和社会组织管理。提升应急联动和防灾减灾救灾能力。扎实开展“七五”普法。

维护社会和谐稳定。加快实施“雪亮工程”，深入推进“守护平安”系列活动，依法打击和惩治黄赌毒黑拐骗等违法犯罪活动，提升平安建设水平。加强信访法治化建设，巩固提高积案化解成果。总结运用十九大维稳安保工作经验，全面开展社会稳定风险评估，做好预测预警预防。完善矛盾纠纷排查化解机制，推进四级综治中心标准化、信息化建设，建立社会心理服务体系。健全信用制度，提升全社会诚信意识。

（八）切实保障和改善民生，不断增强人民群众获得感。坚持以实施民生工程为重要抓手，加强社会保障体系建设，补齐社会事业短板，推进基本公共服务均等化。

加强就业和社会保障。扎实推进就业优先战略，完善积极就业政策，深入实施高校毕业生帮扶计划和就业扶持民生工程，统筹抓好农村转移劳动力、城镇困难人员、退役军人等各类群体就业，城镇新增就业2.9万人。全面落实全民参保计划，推进城乡居民基本医疗保险、基本养老保险等民生工程，“五险”参保190万人次以上。统筹城乡社会救助体系，健全农村留守儿童和妇女、老年人关爱服务体系，发展残疾人事业，加强残疾康复服务。

推进健康宿州建设。大力发展卫生计生事业，实施全民健康保障工程、全民健康促进计划、慢性病综合防控策略，加快推进“智慧医疗”建设，深入开展“健康进万家”活动。深化医药卫生体制综合改革，强化医联体、医共体和家庭医生签约服务等分级诊疗制度建设。巩固提升卫生计生队伍转型成果。深入实施医疗卫生服务体系“411”计划。加强“四型”医院创建，实施卫生人才队伍建设“三大计划”。传承发展中医药事业，深入贯彻中医药法。大力发展健康产业。

统筹社会事业发展。大力发展文化事业、文化产业，加强文化遗产保护和利用，深入实施文化惠民工程，扶持书画、戏曲等文艺事业发展。深化教育体制机制改革，加强学前教育规范管理，强化公办幼儿园示范作用；扩大优质义务教育资源覆盖面；多样化发展高中教育；积极发展现代职业教育，加快市职教园区和县区中职学校建设。加强校舍维修改造，补齐教育短板，提升教育教学质量。加强师德师风建设，培养高素质教师队伍。加强国防教育，增强全民国防观念。实施全民科学素质行动计划。开展好第四次全国经济普查。发展体育事业，加快体育场馆建设，打造城市社区15分钟健身圈，积极备战省第十四届运动会。支持人民团体发挥桥梁纽带作用，加强民族宗教、外事侨务、广播电视、地方志、档案、防震减灾、红十字、妇女儿童、对台、气象等工作。

四、切实加强新时代政府自身建设

新时代对新一届政府工作提出了新的更高要求，我们将深入贯彻习近平新时代中国特色社会主义思想，以新发展理念为引领，以“红船精神”为动力，加强和改进政府自身建设，不断提升政府治理能力现代化水平。

坚持转变理念，建设笃行有为的担当政府。准确把握新时代新特征，强化新理念新思维，坚决维护党中央权威，把对党忠诚、为党分忧、为党尽职、为民造福作为根本政治担当。按照市委“2+4+2”工作思路，注重统筹兼顾、弹好钢琴，善谋善断、善作善成，做到守土有责、守土尽责。把提高执行力作为重中之重，以实干、实在、实绩为工作导向，以肯干事、能干事、不出事为担当标准，确保各项工作踏石留印、抓铁有痕。健全正向激励和容错纠错机制，严格落实“三个区分开来”，旗帜鲜明地为敢闯敢试者撑腰鼓劲，对慵懒无为者严肃追责，努力营造干事创业、奋发有为的政治生态。

坚持转变职能，建设便捷高效的服务政府。深化简政放权，进一步理顺政府与企业、市场、社会的关系。更多运用市场化、社会化、信息化方式加强服务管理，全面实施“最多跑一次”改革，继续深化“互联网+政务服务”，实现网上办事“键对键、不见面”，服务事项“一号申请、一窗受理、一网通办”，让信息多跑路、群众少跑腿。持续开展“四送一服”双千工程，认真落实各项政策措施，构建“亲”“清”新型政商关系，创优“四最”营商环境，努力打造政策洼地、服务高地。

坚持权责法定，建设依法行政的法治政府。严格遵守宪法法律，全面提高法治思维和依法行政能力，确保政府工作在法治轨道上运行。坚持规章、规范性文件立改废释并举，完善并严格执行重大决策法定程序，落实法律顾问、公职律师制度，全面推行重大执法决定法制审核制度。试行行政复议网上受理，提高仲裁公信力，依法有效化解社会矛盾。自觉接受人大及其常委会监督和政协民主监督，完善12345政府热线平台，健全接受社会监督和舆论监督机制。深入推进政务公开，打造透明政府，让权力阳光运行。

坚持从严治政，建设风清气正的廉洁政府。全面贯彻中央八项规定精神，落实深入推进作风建设的实施细则，突出治理“四风”问题隐形变异新表现，坚决反对形式主义和官僚主义。强化监督检查，紧盯关键节点，严格执纪问责，驰而不息正风肃纪，坚决打赢作风建设攻坚战、持久战。以零容忍态度惩治腐败，坚决按照加强党风廉政建设和反腐败工作总体部署，紧密结合政府工作实际，认真履行“一岗双责”，保持正风反腐高压态势。全面加强反腐倡廉制度建设，健全廉政风险防控机制，推进审计监督全覆盖，严防权力失控、决策失误、行为失范，树立起新一届政府为政清廉、干净干事的良好形象。

各位代表！新蓝图描绘新愿景，新起点开启新征程。让我们更加紧密地团结在以习近平同志为核心的党中央周围，在习近平新时代中国特色社会主义思想指引下，在省委、省政府和市委坚强领导下，不忘初心，牢记使命，锐意开拓，扎实苦干，为建设富强民主文明和谐美丽新宿州而努力奋斗！
